# Study of Concrete Surface Coatings Using Thermosensitive Fluorescent Microcapsules Capable of Indicating Damage

**DOI:** 10.3390/gels8090583

**Published:** 2022-09-14

**Authors:** Haohui Zhang, Qing Wang, Yao Li, Yayun Zhao

**Affiliations:** 1 College of Civil Engineering and Architecture, Shandong University of Science and Technology, Qingdao 266590, China; 2 College of Mechanical and Architectural Engineering, Taishan University, Tai’an 271000, China

**Keywords:** microcapsules, thermosensitive fluorescent, damage indicating, micro-cracks, cement-based materials, concrete surface coating

## Abstract

A new type of concrete surface gel coating using thermosensitive fluorescent (TSF) microcapsules was proposed to monitor micro-cracks of cement-based materials. The gel materials can adhere other materials, and the incorporation of microcapsules into the gel coating can be cured on various structural surfaces. Zinc sulfide and phenyl acetate were encapsulated into a polymethyl methacrylate shell to prepare the TSF microcapsules by a solvent evaporation method. When micro-cracks are generated on the surface of the gel coating, the ruptured TSF microcapsules burst out, fill the damaged area, and then emit fluorescence after being excited at ambient temperature. It was found that the brightness of the fluorescence increased with increasing temperature from 80–110 °C. When the concentration of TSF microcapsules was 15% of the mass of the gel coating, the cement-based damage-sensing material had sufficient damage-indicating effects, and the fluorescence brightness of the crack location remained even after a long time. It is expected that this study will provide an effective and intuitive method for crack location detection of cement-based materials.

## 1. Introduction

Cement-based materials are widely used in infrastructure construction because of their high compressive strength, low cost, and convenient assembly [1]. However, cement-based materials are prone to cracks due to their low tensile strength and the influence of temperature stress [2]. The problem of cracks in cement-based materials has received widespread attention [3,4,5,6]. The development of cracks will reduce the service life of the materials and may even lead to failure of the entire structure [7,8]. If micro-cracks can be detected at early stages of crack development, the continuous development of micro-cracks can be avoided, and the safety of the structure can also be improved [9].

At present, many crack detection methods based on sensors or detectors have been studied. For example, optical fiber sensors [10,11,12,13], flexible strain sensors [14], piezoelectric ceramic sensors [15,16], and acoustic emission sensors [17,18] have all been used to detect the development of cracks. However, these sensors require data acquisition equipment, which is complicated and expensive. To find a simple and low-cost damage detection method, researchers have proposed fluorescent crack sensors based on cinnamate [19], dimericstyrylpyrylium [20], and anthracene [21]. After the cracks aregenerated, fluorescence can be emitted to monitor the damaged part. However, it was found that the sensor structure was unstable when exposed to the external environment, which affected the fluorescence effect.

To prevent the dye from being damp or oxidized and decomposed, a damage-monitoring sensor based on microcapsules has been proposed [22,23,24]. This microcapsule sensor can coat dyes to maintain the stability of the core material. The cracks cause the microcapsules to burst and the core material to flow out, highlighting the damaged part through color change or fluorescence emission. Zheng et al. [25] encapsulated crystal violet lactone dye in polymethyl methacrylate as the shell material to prepare microcapsules by a solvent evaporation method, and a layer of silicon dioxide was adhered onto the polymethyl methacrylate shell. When the microcapsules were ruptured, the core material crystal violet lactone reacted with the silica attached to the shell material to show a blue color, so as to indicate the damaged site. Because the color change was not obvious and it was difficult to observe small damages in a dark environment, Lavrenova et al. [26] prepared polyurea-formaldehyde resin microcapsules with toluene and hexamethylbenzene as donors and trichlorquinone as acceptors by an in-situ polymerization method. When the two types of microcapsules ruptured, the donor and the acceptor bodies combined to form a charge transfer complex, which caused a color change from yellow to red. However, since the donor and the acceptor existed in the two microcapsules, the uneven distribution of the microcapsules in the matrix resulted in a low probability of binding. Therefore, the expression effect on the damage site was not obvious. Odom et al. [27] encapsulated 1,3,5,7-cyclooctatetraene in polyurea formaldehyde and a polyurethane shell to fabricate microcapsules by an in-situ emulsification polycondensation method, where the microcapsules and catalyst were dispersed in the coating. When the microcapsules were ruptured, 1,3,5,7-cyclooctatetraene could react with a Grubbs-Love ruthenium catalyst to show a color change from almost colorless to orange-red to deep purple. However, the catalyst may be deactivated in the matrix, resulting in insufficient reaction and poor color development. Therefore, it is necessary to propose a new type of microcapsule for the damage monitoring of cracks.

In this paper, a new type of concrete surface gel coating using thermosensitive fluorescent (TSF) microcapsules is proposed to monitor micro-cracks of cement-based materials. This thermosensitive fluorescent microcapsule can be excited by temperature to emit strong fluorescence, and the fluorescence brightness becomes stronger with increasing temperature. The gel materials can adhere to other materials, and the incorporation of microcapsules into the gel coating can be cured on various structural surfaces. The morphology, chemical structure, and thermal stability of the TSF microcapsules were studied by scanning electron microscopy (SEM), Fourier Transform infrared spectroscopy (FT-IR), and a thermogravimetric analyzer(TGA). The effect of stirring speed on the particle size of the TSF microcapsules was explored by nano measurer, and the effect of agitation speed on the size of TSF microcapsules was studied. The fluorescence brightness of TSF microcapsules under excitation at different temperatures was explored. A stable/transient fluorescence spectrometer was used to study the ultraviolet excitation wavelength of the TSF microcapsules and the fluorescence emission intensity at different temperatures after excitation at this ultraviolet wavelength. TSF microcapsules with varying contents were mixed into the gel coating to investigate the fluorescence indicating effect after artificially causing the cracks under heating excitation. It is expected that the proposed method based on TSF microcapsules may provide a new way to monitor the micro-cracks of cement-based materials.

## 2. Experiments

### 2.1. Materials

Phenyl acetate (99%) was purchased from McLean Biochemical Technology Co., Ltd. (Shanghai, China). Zinc sulfide phosphor was purchased from Xiucai Chemical Co., Ltd. (Foshan, China). Polymethyl methacrylate (PMMA, 100 mesh) was purchased from Zhongxin Plastic Co., Ltd. (Taizhou, China). Dichloromethane (≥99.5%) was purchased from Kelon Chemical Co., Ltd. (Chengdu, China). Polyvinyl alcohol (PVA, 100 mesh) was purchased from Usolf Chemical Technology Co., Ltd. (Qingdao, China). Epoxy resin E-51 and T-31 curing agents were purchased from Haozhuo Material Technology Co., Ltd. (Nanjing, China).

### 2.2. Preparation of TSF Microcapsules

Seventy milliliters of water was added into abeaker and 1.6 g of PVA emulsifier was slowly added while heating to 40 °C. Then, the temperature was raised to 80 °C at 1000 rpm/min to fully dissolve it by using a magnetic stirrer (85-2A, Jintan Instrument, Changzhou, China). It was cooled to room temperature and used as the emulsifier solution. One gram of PMMA was added into 30 g of dichloromethane solution and stirred with a glass rod for full dissolution. Then, 0.25 g of zinc sulfide phosphor was added into 2 g of phenyl acetate solution and mixed together using a magnetic stirrer at agitation speeds of 400, 700, 1000, and 1300 rpm/min, respectively. They were stirred for 5 h at 40 °C to evaporate the dichloromethane. The reaction mixture was allowed to stand for 30 min, and then the supernatant was poured out. It was rinsed with deionized water four times to obtain microcapsule precipitation, and dried microcapsules were generated after 24 h at room temperature. The dry microcapsules are TSF microcapsules containing zinc sulfide and phenyl acetate in the PMMA shell.

### 2.3. Preparation of Cement-Based Damage Sensing Materials

The mortar test block was made according to a ratio of cement: sand: water—1:1:0.5. The stirring speed can control the particle size of TSF microcapsules. In this paper, considering the function of the microcapsules, we selected TSF microcapsules prepared at 1000 rpm/min and added them at 5%, 10%, 15%, and 20% of the total mass of epoxy resin and curing agent to prepare the damage detection coating. The ratio of epoxy resin and curing agent was 3:1. It was applied on the surface of the mortar test block and cured at room temperature for 48 h, and then a gray shading coating was sprayed onto it.

### 2.4. Characterization and Performance Test

The morphology and shape of the TSF microcapsules were investigated by scanning electron microscope (SEM; APERO, Saint Paul, MN, USA) at a voltage of 1.00 kV. Anion sputtering apparatus (GVC-1000, Microhezao, Shenzhen, China) was used to place a conductive gold film on the surface of the microcapsule sample for better imaging quality. Nano measurer software was used to select no less than 300 microcapsules for measurement, and the particle size distribution of TSF microcapsules was analyzed at different stirring rates. Fourier Transform infrared spectroscopy (FT-IR; Nicolet iS50, Austin, TX, USA) was used to analyze the chemical structure of the TSF microcapsules. The sample was mixed with dry KBr powder to make tablets. The spectral range was from 4000–500 cm^−1^. A thermogravimetric analyzer (TGA; STA449C, NETZSCH, Selb, Germany) was used to characterize the thermal stability of the TSF microcapsules. The test protocol was to heat from 30 °C to 600 °C at a rate of 10 °C/min. The flow rate of nitrogen was 20 mL/min, and liquid nitrogen was used as a cooling source. Excitation spectra and variable temperature fluorescence emission spectra were studied by fluorescence spectrophotometer (F-7100, Hitachi, Taito-ku, Tokyo, Japan). The variable temperature fluorescence emission spectrum was determined in the emission range of 400–700 nm. The sample was illuminated by a xenon arc lamp with a excitation energy of 380 nm. The TSF microcapsules were placed into a glass tube and immersed in oil solution at temperatures of 80 °C, 90 °C, 100 °C and 110 °C, respectively, to observe the fluorescence effect. A layer of epoxy resin coating containing 5%, 10%, 15% and 20% TSF microcapsules was coated on the surface of the mortar test block to observe its damage indication effect. Then, a layer of shading layer was sprayed onto it. A blade was used to create cracks, and then samples were placed in an oil solution at 100 °C to observe the fluorescence effect. A digital microscope (AOSVI, AO-AF26C, Nanjing, China) was used to photograph the crack width. The normal mode and long exposure mode of a digital camera (EOS 850D, Canon, Ōta, Tokyo, Japan) were used to shoot the damage indicator effect of the sample in a dark environment.

## 3. Results and Discussion

### 3.1. Morphology Characterization of TSF Microcapsules

**Figure 1:**
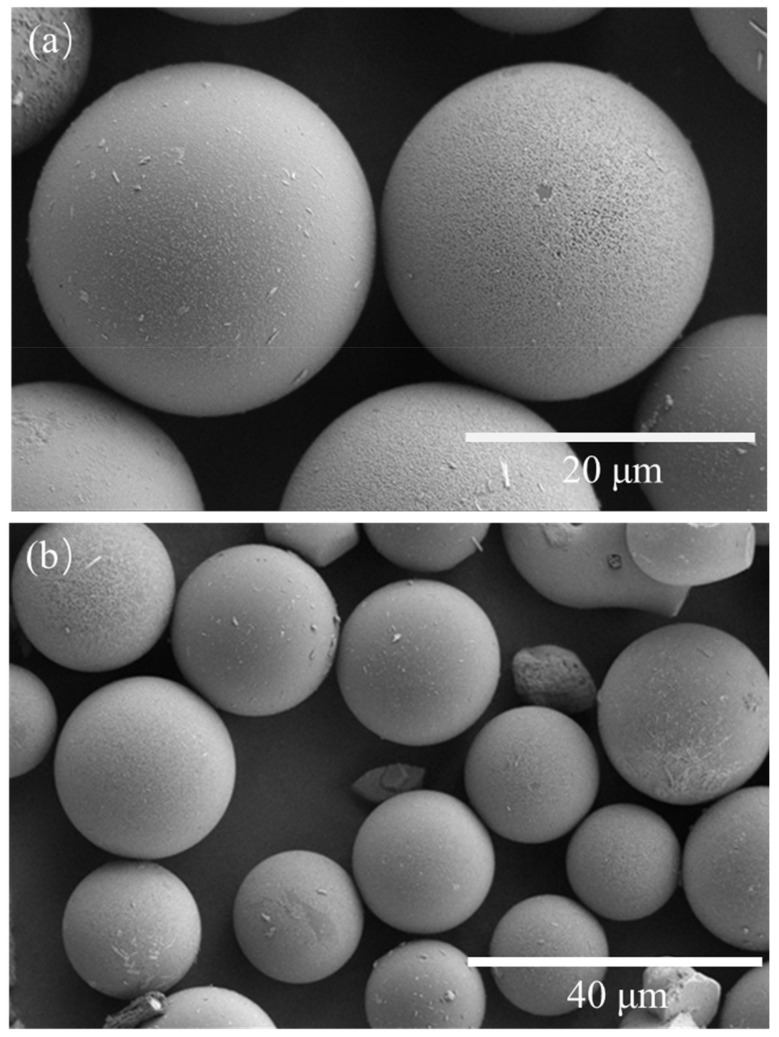
SEM images of TSF microcapsules at (**a**) 8000 times magnification and (**b**) 4000 times magnification.

The shape and morphology of the synthesized TSF microcapsules were evaluated utilizing SEM micrographs. Figure 1 shows the SEM micrographs of the TSF microcapsules under different magnifications. The TSF microcapsules prepared at an agitation speed of 1000 rpm/min were used for shooting. It can be seen from Figure 1a that the TSF microcapsules are spherical in shape, and the outer wall is smooth without holes or cracks. Figure 1b shows that the particle size distribution of the TSF microcapsules are uniform, most of which are distributed between 10 μm and 20 μm. There is no adhesion or agglomeration between the microcapsules, which is conducive to the uniform distribution within the coating.

### 3.2. Analysis of Chemical Composition of TSF Microcapsules

**Figure 2:**
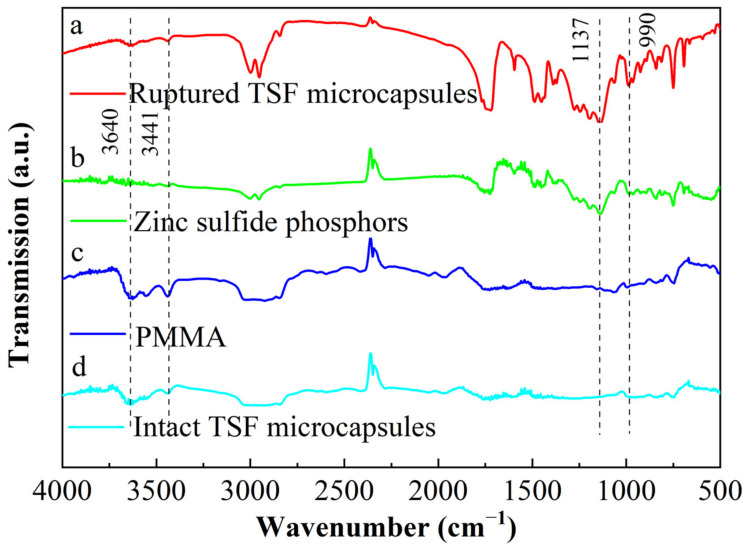
Fourier infrared spectrum: (a) ruptured TSF microcapsules; (b) zinc sulfide phosphors; (c) PMMA; (d) intact TSF microcapsules.

The microencapsulation of the core material was investigated by FT-IR. Figure 2 shows the FT-IR spectra of ruptured TSF microcapsules (curve a), zinc sulfide phosphors (curve b), PMMA (curve c), and intact TSF microcapsules (curve d). The appearance of transmittance peaks in the ruptured TSF microcapsules, PMMA, and intact TSF microcapsules at 3640 cm^−1^ and 3441 cm^−1^ are ascribed to the O-H stretching vibration absorption peak in the PMMA matrix [28]. This indicates that the characteristic peaks of PMMA shell material can be seen in both intact TSF microcapsules and ruptured TSF microcapsules. The peaks recorded at 1137 and 990 cm^−1^ in zinc sulfide phosphors are assigned to the main characteristic absorption peaks of zinc sulfide [29]. These peaks can also be found in ruptured TSF microcapsules, but these peaks are not found in intact TSF microcapsules. This shows that the characteristic peak of the core material can be observed in the broken TSF microcapsules, but the characteristic peak of the core material is not found in the intact TSF microcapsules. From the above analysis, it can be concluded that the core material is successfully microencapsulated.

### 3.3. Particle Size Distribution of TSF Microcapsules

**Figure 3:**
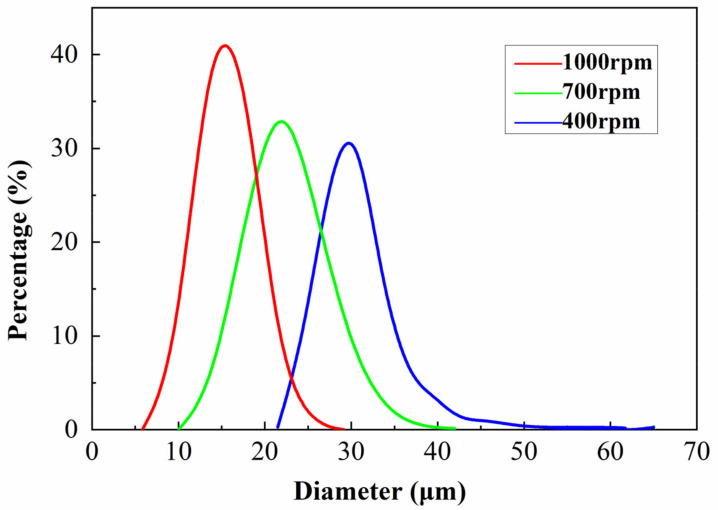
Particle size distribution of TSF microcapsules under different stirring speeds.

The particle size distribution of TSF microcapsules prepared at agitation speeds of 400, 700, and 1000 rpm/min is shown in Figure 3. The core material-coated microcapsules were not formed at an agitation speed of 1300 rpm/min. This is due to excessive shear force at 1300 rpm, where the viscosity of the emulsion decreases and the stability of the emulsion deteriorates [30]. Well-coated microcapsules could be formed at speeds of 400–1000 rpm/min. The particle diameters of the TSF microcapsules fabricated at 400 rpm/min, 700 rpm/min, and 1000 rpm/min are approximately in the range of 20–60 μm, 10–40 μm, and 5–30 μm, and the main particle sizes are 30 μm, 20 μm, and 15 μm, respectively. It can be seen from Figure 3 that as the agitation speed increases, the particle size of the TSF microcapsules decreases and the particle size distribution becomes narrow. This is consistent with the research results of Zheng et al. [25]. As the agitation speed increases, the shearing force also increases, which results in a smaller microcapsule particle size [31]. This result also indicates that the particle size of the TSF microcapsules can be controlled by the stirring speed.

### 3.4. Thermal Stability Analysis of TSF Microcapsules

**Figure 4:**
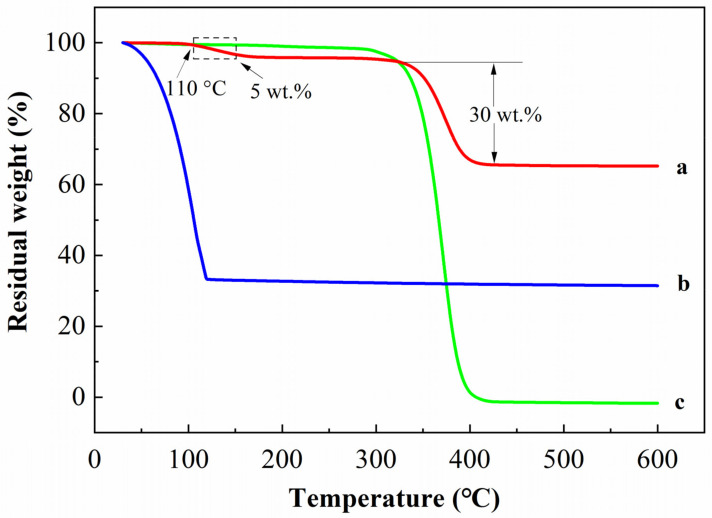
Thermogravimetric analysis: (a) TSF microcapsules; (b) zinc sulfide phosphors and phenyl acetate; (c) PMMA.

The thermal stability of TSF microcapsules is critical to their practical application. Figure 4 shows the TGA results of TSF microcapsules, zinc sulfide phosphors, phenyl acetate core materials, and PMMA shell materials. It can be seen from the TGA of TSF microcapsules and PMMA shell materials that there is weight loss at 350–400 °C, which is due to the thermal decomposition of the PMMA shell material, which accounts for 30% of the mass of the microcapsule. Analyzing the TGA of the TSF microcapsules shows a weight loss of 5 wt.% at 110 °C. This thermal decomposition is also observed at this temperature in the TGA of core materials, which is due to the decomposition of phenyl acetate in the core material. From the TGA results of TSF microcapsules and core materials, the temperature at which the TSF microcapsules begin to decompose increases relative to the temperature at which the core material begins to decompose. This shows that microencapsulation of the core material can improve its thermal stability. When the microcapsule temperature is greater than 400 °C, only the zinc sulfide phosphor is left, and there is no quality loss between 400 °C and 600 °C, which shows that the zinc sulfide phosphor has good heat resistance. In addition, the content of the core material accounts for 70% of the mass of the microcapsules, which can more effectively indicate damage. The TGA results demonstrate that PMMA successfully coats the zinc sulfide phosphor, and the thermal stability of the core material is significantly improved after coating.

### 3.5. Fluorescence Effects of TSF Microcapsules

**Figure 5:**
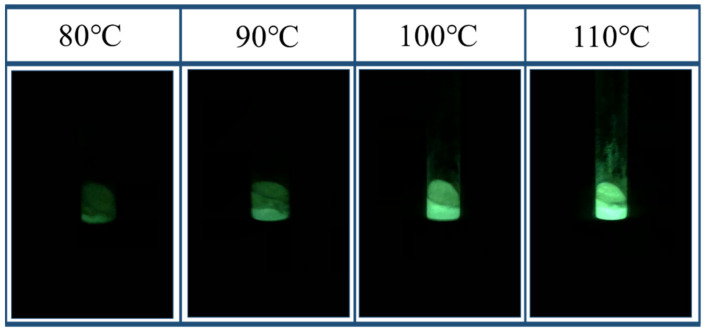
Fluorescence effect of TSF microcapsules excited by different temperatures.

The fluorescence effect of TSF microcapsules under excitation at different temperatures of 80 °C, 90 °C, 100 °C, and 110 °C is shown in Figure 5. The TSF microcapsules begin to lose weight at 110 °C, and the evaporation of phenyl acetate in the microcapsules causes the fluidity of the core material to decrease [32]. As a result, the zinc sulfide phosphors cannot be fully distributed in the micro-cracks. Therefore, the maximum thermal reaction temperature of the TSF microcapsules is set to 110 °C. The oil solution was heated to 80 °C, 90 °C, 100 °C and 110 °C, respectively, and then the glass tube containing the TSF microcapsules was placed into it to observe the fluorescence effects. Compared with the damage indicating microcapsule prepared by Credico et al. [33] which can highlight the color change of the damaged area only under the excitation of ultraviolet light, the thermosensitive fluorescent microcapsules proposed in this paper can be excited not only by ultraviolet light, but also by temperature to emit strong fluorescence. The fluorescence brightness becomes stronger with the increase of temperature. It can be seen from Figure 5 that as the temperature increases, the fluorescence brightness increases. This is mainly because the electrons stored in the defect energy level of the zinc sulfide phosphor are converted into an excited state due to thermal disturbance, and then transition back to the ground state to emit light.

**Figure 6:**
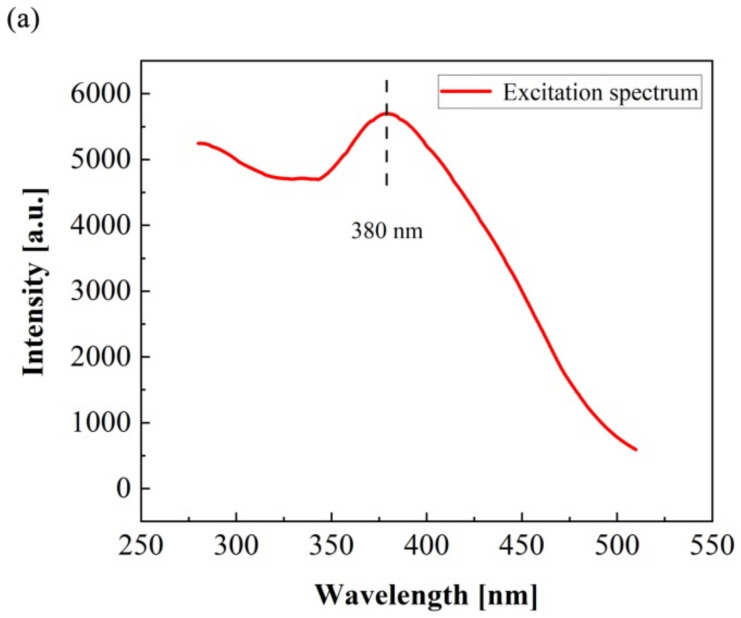

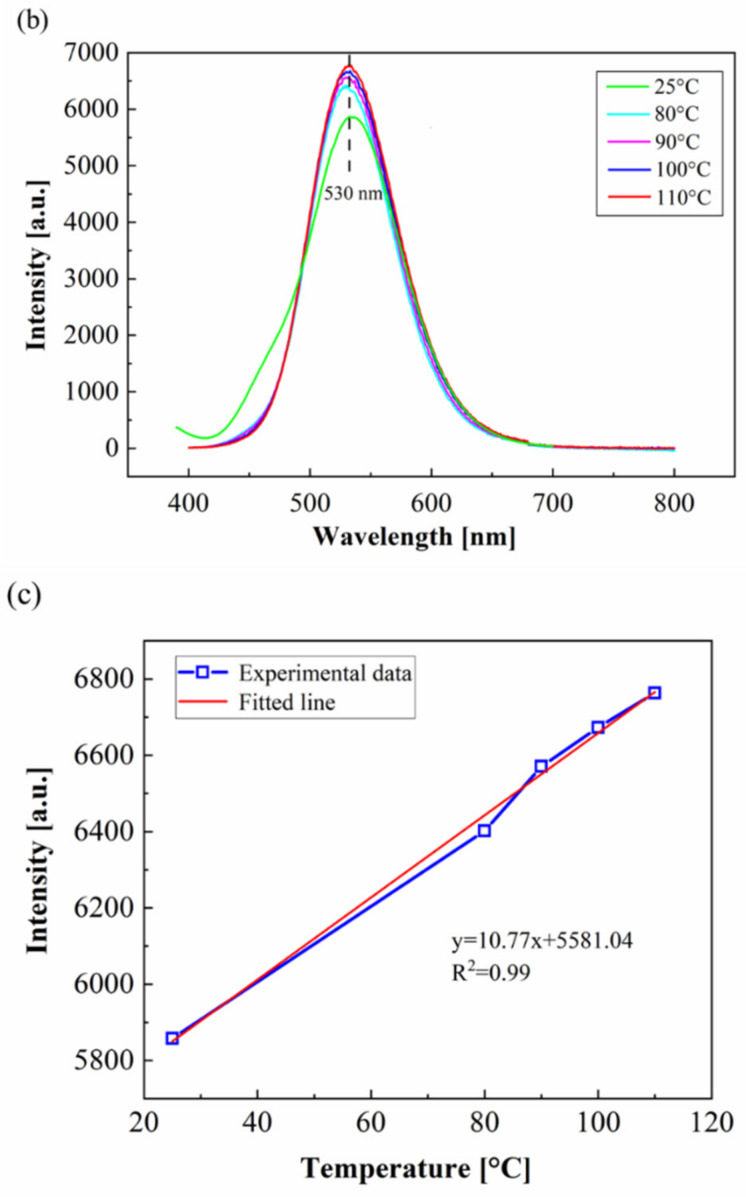
Fluorescence spectra of TSF microcapsules. (**a**) Excitation spectrum, (**b**) variable temperature fluorescence emission spectrum, (**c**) the strongest fluorescence intensity value at different temperatures.

Figure 6 shows the excitation spectrum, the variable temperature fluorescence emission spectrum, and the strongest fluorescence intensity at different temperatures of the TSF microcapsules, respectively. It can be seen from Figure 6a that the TSF microcapsules have a strong excitation peak at a wavelength of 380 nm. This indicates that the TSF microcapsules are excited with 380 nm ultraviolet light to obtain the corresponding variable temperature fluorescence emission spectra. The temperatures are set to 25 °C, 80 °C, 90 °C, 100 °C, and 110 °C, respectively. As shown in Figure 6b, the TSF microcapsules produce the strongest fluorescence intensity at a wavelength of 530 nm at different temperatures. The peak at 530 nm is due to the defect or surface states within the band gap [34]. It can be seen from Figure 6c that the fluorescence intensity of the TSF microcapsules shows a linear growth trend with increasing temperature. The maximum fluorescence intensity value emitted by the TSF microcapsules at 110 °C is 15.4% higher than the maximum fluorescence intensity emitted at 25 °C. These results indicate that the TSF microcapsules are excited by high temperature and ultraviolet light to fluoresce, and the fluorescence intensity strengthens as the temperature rises.

### 3.6. Evaluation of Crack Damage Indication

**Figure 7:**
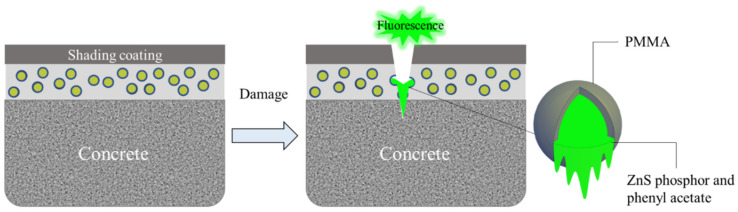
Schematic diagram of damage indication.

**Figure 8:**
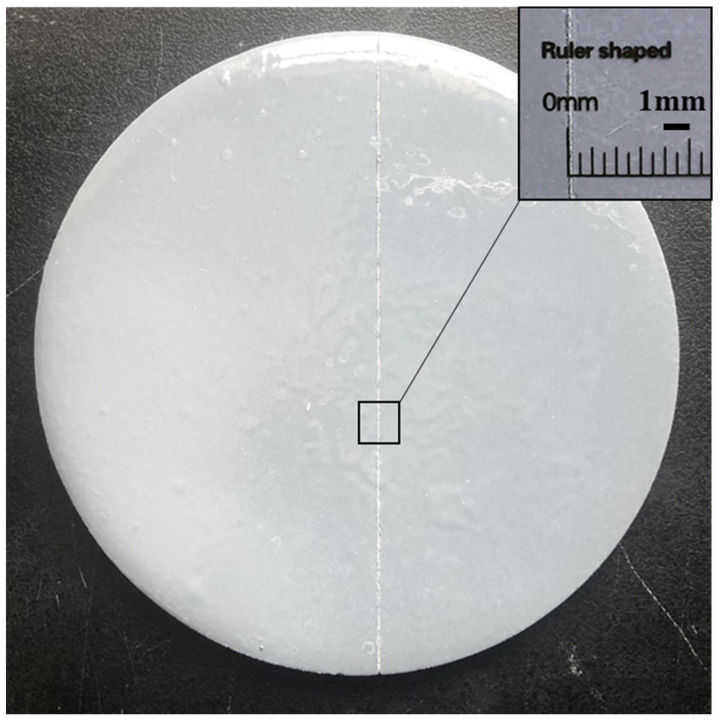
Damage samples of the artificially-generated crack.

**Figure 9:**
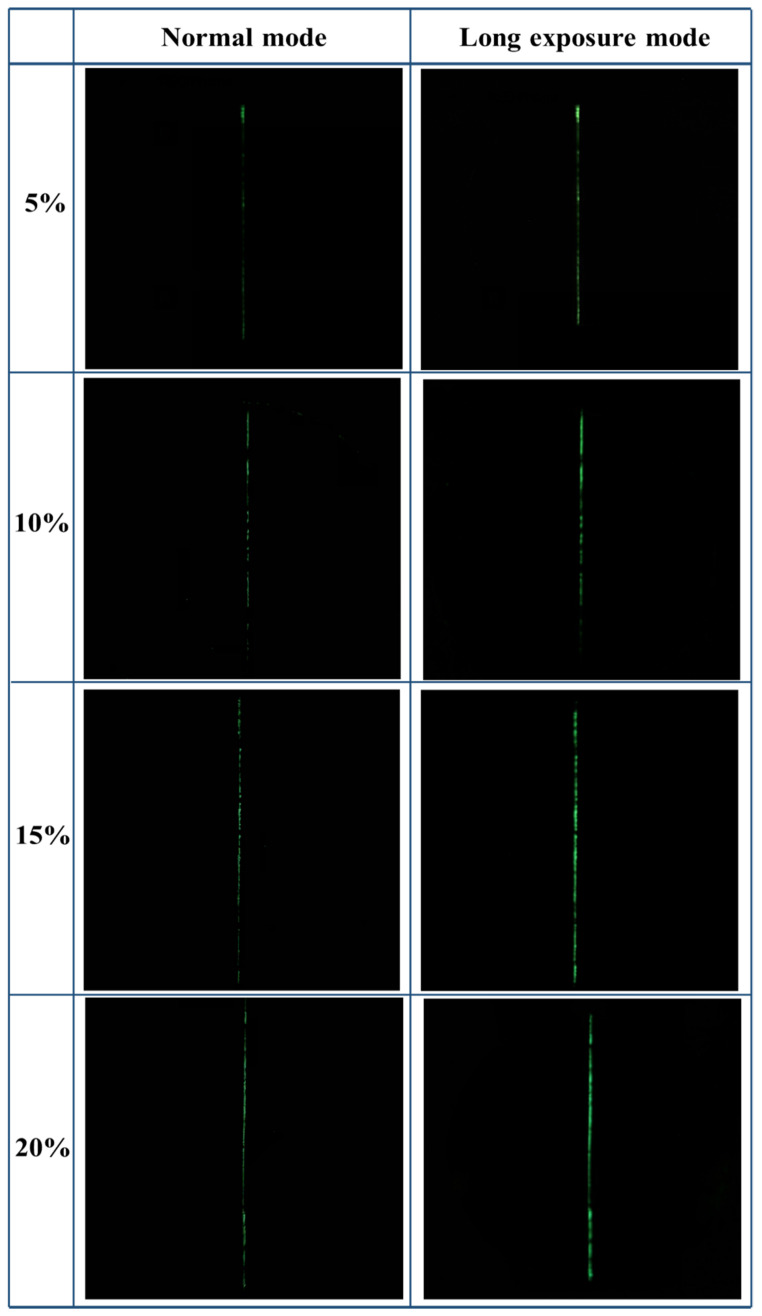
The damage indicating effect of coatings with different TSF microcapsule contents taken in normal mode and long exposure mode.

The damage indication concept of cement-based damage-sensing materials is illustrated in Figure 7. A layer of epoxy resin coating containing TSF microcapsules and a light-shielding coating are coated on the surface of cement-based materials to prepare the cement-based damage-sensing materials. When the micro-cracks develop on the surface of the material, the microcapsules will be ruptured. The fluorescent core liquid will flow out, fill the damaged area, and then emit fluorescence after being excited by temperature. The damage sample after the crack is artificially made is shown in Figure 8. It can be seen from Figure 8 that the crack width is about 0.1 mm. Figure 9 shows the damage indicating effect of different contents of TSF microcapsule coatings in normal mode and long exposure mode. Epoxy resin coatings containing 5%, 10%, 15%, and 20% TSF microcapsule contents were prepared, respectively. The coating was scratched and damaged with a blade and then placed in a 100 °C oil solution to observe the damage indicating effect. From Figure 9, it can be observed that in a dark environment, as the content of TSF microcapsules in the coating increases, the fluorescence intensity becomes strong. This is because the density of the TSF microcapsules increases, which releases more fluorescent fluid under capillary action. In coatings containing 5% and 10% TSF microcapsules, the fluorescent fluid cannot completely fill the cracks, resulting in insignificant fluorescent effects. When the content of TSF microcapsules incorporated into the coating is 15% and 20%, the damage indication effect is better than that of 5% and 10% microcapsules. The damage indication effect picture in the dark environment shot in long exposure mode is stronger than the fluorescent brightness observed by the naked eye. This method can be used to observe micro-crack damage not easily observed with the naked eye.

## 4. Conclusions

This paper proposed a new type of TSF microcapsule that can be used for damage detection of cement-based materials. The TSF microcapsules were spherical in shape, and the outer wall was smooth without holes and cracks. The thermal stability of the core material was improved after microencapsulation by thermogravimetric analysis, and it can withstand temperatures up to 110 °C. The particle size analysis showed that the particle size of the TSF microcapsules became smaller and the distribution narrowed with increasing stirring speed. The TSF microcapsules were excited by both ultraviolet light and temperature, and the fluorescence intensity of the TSF microcapsules strengthened with increasing temperature in the range of 80–110 °C. As the content of TSF microcapsules incorporated into the coating increased, the damage indicating effect became obvious. When the content of TSF microcapsules is 15% of the mass of the epoxy resin coating, the cement-based damage sensing material has sufficient damage indicating effects. By using the long exposure mode to shoot the damaged part, it was possible to monitor the small micro-cracks that were not easily observed in a dark environment.

## Data Availability

The study did not report any data.
